# Peripheral blood inflammatory ratios predict efficacy and toxicity of CAR-T cell immunotherapy in relapsed/refractory multiple myeloma

**DOI:** 10.3389/fimmu.2026.1752235

**Published:** 2026-02-25

**Authors:** Peng Xu, Can Huang, Yao Liu, Ke Ji, Wei Dai, Yang Liu, Zhi-Ling Yan, Huan-Xin Zhang, Chong Chen, Jiang Cao, Qing-Yun Wu

**Affiliations:** 1 Blood Disease Institute, Key Laboratory of Bone Marrow Stem Cell, Xuzhou Medical University, Xuzhou, China; 2 Department of Hematology, The Affiliated Hospital of Xuzhou Medical University, Xuzhou, China; 3 Department of Gastroenterology, The Affiliated Hospital of Xuzhou Medical University, Xuzhou, China; 4 Department of Pharmacy, Xuzhou Medical University Affiliated Hospital Sihong Branch, Xuzhou, China; 5 Suzhou Hongci Hematology Hospital, Suzhou, China

**Keywords:** chimeric antigen receptor T cell, multiple myeloma, peripheral blood inflammatory ratios, prognostic predictor, relapsed/refractory

## Abstract

**Background aim:**

Despite the remarkable efficacy of chimeric antigen receptor T-cell (CAR-T) therapy in relapsed/refractory multiple myeloma (R/R MM), treatment response and toxicity exhibit considerable heterogeneity. This study aimed to evaluate the prognostic significance of baseline peripheral blood inflammatory ratios—namely, the neutrophil-to-lymphocyte ratio (NLR), monocyte-to-lymphocyte ratio (MLR), and platelet-to-lymphocyte ratio (PLR)—in patients with R/R MM receiving CAR-T therapy, and to develop an integrated prognostic index based on these parameters.

**Methods:**

We conducted a retrospective analysis of 197 R/R MM patients who received CAR-T therapy. The optimal cut-off values for NLR, MLR, and PLR were determined using receiver operating characteristic (ROC) curve analysis. Associations between these ratios and treatment efficacy, CAR transgene expansion, cytokine release syndrome (CRS), and progression-free survival (PFS) were evaluated. A composite Cellular Inflammatory Prognostic Index (CIPI) integrating NLR, MLR, and PLR was developed to assess prognostic stratification.

**Results:**

Optimal cut-offs for NLR, MLR, and PLR were 2.55, 0.35, and 145, respectively. Patients with low baseline inflammatory ratios exhibited significantly higher CAR transgene expansion and were associated with better treatment responses than that of patients with high baseline inflammatory ratios. The low NLR group showed a superior objective response rate (93.8% vs. 81.2%, *p* = 0.037) and a longer median PFS was observed in the low NLR group compared with the high NLR group (18.6 vs. 10.9 months, p = 0.0012). Elevated inflammatory ratios correlated with high peak levels of IL-6 and ferritin and an increased incidence of severe CRS (≥ grade 3). The CIPI score effectively stratified patients into low-, intermediate-, and high-risk groups with distinct PFS (median PFS: 18.9, 13.8, and 5.1 months, respectively; *p* < 0.0001). Multivariate analysis confirmed that the CIPI score was an independent prognostic factor for PFS, along with high tumor burden.

**Conclusion:**

Baseline peripheral blood inflammatory ratios are closely associated with CAR-T cell efficacy and CRS severity in R/R MM patients receiving CAR-T therapy. The CIPI score represents a simple and reproducible prognostic biomarker that may help individualized risk stratification and inform treatment optimization in CAR-T therapy.

## Introduction

Multiple myeloma (MM), the second most common hematologic malignancy, is a malignant disease arising from the clonal proliferation of plasma cells (1, 2). Although the application of novel immunomodulatory agents, proteasome inhibitors, and monoclonal antibodies has significantly improved patients’ outcomes, most patients ultimately develop a relapsed or refractory (R/R) state (3–5). In recent years, B-cell maturation antigen (BCMA)-targeted chimeric antigen receptor T-cell (CAR-T) therapy has demonstrated remarkable efficacy in treating R/R MM, significantly improving deep response rates and prolonging progression-free survival (6–8). However, not all patients derive lasting benefit from this treatment; some experience disease progression or relapse shortly after CAR-T infusion, indicating significant heterogeneity in treatment response (9, 10). Furthermore, CAR-T therapy-related toxicities, such as cytokine release syndrome (CRS) and immune effector cell- associated neurotoxicity syndrome (ICANS), limit its broad application (10, 11). Therefore, identifying biomarkers that can accurately predict the efficacy and toxicity of CAR-T therapy is of great clinical importance for patient selection, individualized treatment strategy formulation, and prognosis improvement.

Previous studies explored various factors influencing the efficacy and safety of CAR-T therapy. Que et al. found that patients who received six or more prior lines of therapy had shorter progression-free survival (PFS) and overall survival (OS) than that of patients not received, but when extramedullary disease (EMD) was included in the multivariate analysis, the predictive value of “≥ 6 lines of therapy” disappeared, leaving EMD as the only statistically significant independent prognostic factor (12). Zhang et al. reported that EMD, light chain MM, high-risk cytogenetic features, and more than three prior lines of therapy were all independent risk factors for PFS after CAR-T therapy (13). Regarding safety, inflammatory markers such as ferritin, C-reactive protein (CRP), and interleukin-6 (IL-6) have also been confirmed to be closely associated with the occurrence of CRS during treatment (14, 15).

In recent years, peripheral blood inflammatory ratios—such as the neutrophil-to- lymphocyte ratio (NLR), monocyte-to-lymphocyte ratio (MLR), and platelet-to- lymphocyte ratio (PLR)—have gained attention due to their accessibility, low cost, and ability to reflect systemic inflammation and immune status (16, 17). As established biomarkers of prognosis in solid tumors and hematologic malignancies, elevated ratios correlate with enhanced pro-inflammatory activity and impaired anti-tumor immune function (18–20). Our center previously studies demonstrated that the high baseline absolute lymphocyte count significantly predicted improved response rates and longer survival in patients with R/R diffuse large B-cell lymphoma (DLBCL) receiving CAR-T therapy (21). However, in the context of CAR-T therapy for R/R MM, the predictive roles of baseline NLR, MLR, and PLR for treatment efficacy and toxicities like CRS remain unclear.

Therefore, this study retrospectively analyzed the clinical data of 197 R/R MM patients who received CAR-T therapy, aiming to investigate associations between baseline NLR, MLR, and PLR with patients’ clinical characteristics, treatment response, PFS, and overall risk of CRS. Additionally, we developed a comprehensive Cellular Inflammatory Prognostic Index (CIPI) to assess its potential utility in prognostic stratification of R/R MM patients receiving CAR-T therapy.

## Patients and methods

### Study population

This study was a retrospective analysis that consecutively enrolled 197 R/R MM patients treated at the Affiliated Hospital of Xuzhou Medical University between June 2019 and February 2024. All patients received BCMA-targeted CAR-T cell therapy, specifically involving either (1): single-target BCMA CAR-T cells, or (2) sequential infusion of BCMA and CD19 CAR-T cells (BCMA+CD19). All CAR−T products were autologous and manufactured in−house. Briefly, peripheral blood mononuclear cells were obtained from subjects by leukapheresis, and CD3^+^ T lymphocytes were isolated using anti−CD3 immunomagnetic beads. A lentiviral vector encoding the CAR construct (anti−BCMA single−chain variable fragment [scFv]/4−1BB/CD3ζ; humanized anti−CD19 scFv/4−1BB/CD3ζ; or a bispecific anti−BCMA/anti−CD19 scFv/4−1BB/CD3ζ) was transduced into CD3^+^ T cells and expanded *in vitro*. CAR−T cells were harvested after 10-14 days and cryopreserved until infusion. The study was approved by the hospital’s Ethics Committee and conducted in accordance with the principles of the Declaration of Helsinki. All participants provided written informed consent. The trials were registered at the Chinese Clinical Trial Registry (ChiCTR.org.cn) with registration numbers ChiCTR2000033567, ChiCTR-OIC-17011272, and ChiCTR1900026219. The detailed inclusion and exclusion criteria were consistent with previously published literature (22, 23). All patients received lymphodepleting conditioning with fludarabine (30 mg/m²/day, days -5 to -3) and cyclophosphamide (750 mg/m²/day, day -5) prior to CAR-T infusion.

### Data collection

Patient clinical characteristics at enrollment were collected, including age, sex, MM type, prior treatment history, and cytogenetic abnormalities. Laboratory data were obtained from the electronic medical record system. Baseline complete blood count, lactate dehydrogenase (LDH), and β2-microglobulin (β2-MG) were defined as the most recent test results within 15 days before lymphodepletion. Peak levels of ferritin, C-reactive protein (CRP), interleukin-6 (IL-6) and CAR transgene were recorded from tests within the first month after CAR-T cell infusion.

### Assessment of inflammatory ratios

The neutrophil-to-lymphocyte ratio (NLR), monocyte-to-lymphocyte ratio (MLR), and platelet-to-lymphocyte ratio (PLR) were calculated based on complete blood count results. The optimal cut-off values for these indices were determined using receiver operating characteristic (ROC) curve analysis, maximizing the Youden index. Patients were stratified into high and low groups based on these cut-offs.

### Definitions and follow-up

The severity of CRS and ICANS was retrospectively graded according to the American Society for Transplantation and Cellular Therapy (ASTCT) consensus criteria (24). Response to CAR-T cell therapy in R/R MM patients was assessed based on the International Myeloma Working Group (IMWG) uniform response criteria updated in 2014 (25). PFS was defined as the time from CAR-T cell infusion to disease progression or death from any cause. The objective response rate (ORR) was defined as the proportion of patients achieving a partial response (PR) or better. To address potential confounding effects on baseline inflammatory ratios, we performed a sensitivity analysis incorporating two clinically relevant variables: previous infections, defined as a documented bacterial or viral infection within 30 days prior to lymphodepletion, and previous corticosteroid use, defined as administration of systemic corticosteroids within 7 days before the baseline complete blood count. All patients were followed until June 30, 2024, with follow-up data sourced from inpatient/outpatient medical records and telephone follow-ups. The median follow−up for the entire cohort was 26.2 months (IQR: 16.5-38.4).

### Statistical analysis

Statistical analyses were performed using GraphPad Prism v8.0 software. Continuous variables are presented as median (range), and comparisons between groups were made using the Mann-Whitney U test or t-test. Categorical variables are presented as frequency (percentage), and comparisons between groups were made using the χ² test or Fisher’s exact test. ROC curves were used to determine the optimal cut-off values for NLR, MLR, and PLR in predicting PFS, along with the area under the curve (AUC). Survival analysis was performed using the Kaplan-Meier method, and group comparisons were made using the log-rank test. Univariate and multivariate Cox proportional hazards regression models were used to analyze independent prognostic factors for PFS. All statistical tests were two-sided, with a *p*-value < 0.05 considered statistically significant.

## Results

### Determination of cut-off values and baseline clinical characteristics

This study included 197 R/R MM patients who received CAR-T cell therapy, with a median age of 58 years (range: 34-78), including 109 males (55.3%). Baseline clinical characteristics, including R-ISS stage, prior treatment history, and disease burden, are detailed in **Table 1**. ROC analysis determined the optimal cut-off values for NLR, MLR, and PLR, to be 2.55, 0.35,and 145, respectively. The AUC for NLR was 0.801 (95% CI: 0.732-0.870, *p* <0.001), with a sensitivity of 82.1% and specificity of 74.5%. The AUC for MLR was 0.763 (95% CI: 0.688-0.838, *p* < 0.001), with a sensitivity of 78.3% and specificity of 70.2%. The AUC for PLR was 0.735 (95% CI: 0.674-0.836, *p* < 0.001), with a sensitivity of 76.5% and specificity of 68.1%. These results suggest that all three ratios possess good discriminatory ability (**Supplementary Figure S1**).

**Table 1:** Demographics and baseline clinical characteristics before CAR-T cell therapy.

Variables	Overall (n = 197)	NLR			MLR			PLR		
		≤2.55 (n = 112)	>2.55 (n = 85)	*P*	≤0.35 (n = 120)	>0.35 (n = 77)	*P*	≤145 (n = 114)	>145 (n = 83)	*P*
Age, years, median (range)	58 (34–78)	58 (39-78)	58 (34-72)	0.713	56 (34-72)	58 (37-78)	0.667	58 (34-78)	56 (39-72)	0.729
Gender, Male, no. (%)	109 (55.3)	68 (60.7)	41 (48.2)	0.085	64 (53.3)	45 (58.4)	0.557	59 (51.8)	50 (60.2)	0.249
R-ISS stage III, no. (%)	114 (57.8)	66 (58.9)	48 (56.5)	0.772	70 (58.3)	44 (57.1)	0.883	62 (54.4)	52 (62.7)	0.306
Type of myeloma, IgG	126 (63.9)	74 (66.1)	52 (61.2)	0.549	79 (65.8)	47 (61.0)	0.544	62 (54.4)	52 (62.7)	0.306
Type of CAR-T cell therapy, no. (%)				0.554			0.549			0.302
BCMA	75 (38.1)	45 (40.2)	30 (35.3)		48 (40.0)	27 (35.1)		47 (41.2)	28 (33.7)	
BCMA+CD19	122 (61.9)	67 (59.8)	55 (64.7)		72 (60.0)	50 (64.9)		67 (58.8)	55 (66.3)	
High tumor burden, no. (%)^a^	48 (24.4)	19 (16.9)	29 (34.1)	**0.007**	22 (18.3)	26 (33.8)	**0.017**	23 (20.2)	25 (30.1)	**0.131**
High-risk cytogenetic features, no. (%)^b^	44 (22.3)	21 (18.8)	23 (27.1)	0.172	24 (20.0)	20 (25.9)	0.382	23 (20.2)	21 (25.3)	0.489
Extramedullary lesions, no. (%)^c^	66 (33.5)	29 (25.9)	37 (43.5)	**0.014**	34 (28.3)	32 (41.6)	0.064	25 (21.9)	41 (49.4)	**<0.001**
Previous therapy lines, median (range)	4 (1-14)	4 (2-12)	4 (1-14)	0.109	4 (1-12)	4 (2-14)	0.522	4 (2-14)	4 (1-14)	0.207
Previous HCT, no. (%)	62 (31.5)	30 (26.8)	32 (37.6)	0.122	36 (30.0)	26 (33.8)	0.638	33 (28.9)	29 (34.9)	0.438
LDH, U/L, median (range)	215 (115-2210)	198 (115-1810)	225 (136-2210)	**0.015**	205 (122-1740)	218 (115-2210)	0.334	212 (115-1890)	219 (130-2210)	0.522
β2-MG, ng/ml, median (range)	2860 (870-20000)	2663 (870-20000)	2895 (875-18000)	0.266	2795 (870-18500)	2990 (887-20000)	0.352	2713 (870-20000)	2898 (892-20000)	0.588

BCMA, B-cell maturation antigen; BCMA+ CD19, sequential infusion of BCMA and CD19 CAR-T cells; HCT, stem cell transplantation; R-ISS, Revised International Staging System.

a High tumor burden: defined as ≥ 50% clonal plasma cells or bone marrow plasma cells.

b High-risk cytogenetics was reported by investigators based on fluorescence *in situ* hybridization. A high-risk cytogenetic profile was defined by the presence of the following abnormalities: del(17p), t (4;14), or t (14; 16).

c Extramedullary diseases included tissue masses in extraosseous locations and bone-related plasmacytomas.

Bold type highlights statistically significant results (*p* < 0.05).

Patients were stratified into high and low groups based on these cut-offs for baseline comparison. Results showed that patients in the high NLR and high MLR groups more frequently had high tumor burden (NLR: 34.1% vs. 16.9%, *p* = 0.007; MLR: 33.8% vs. 18.3%, *p* = 0.017) than that of patients with low NLR and MLR groups, while the incidence of extramedullary disease was significantly higher in the high PLR group compared to the low PLR group (49.4% vs. 21.9%, *p* < 0.001). Additionally, LDH levels were significantly higher in the high NLR group compared with the low NLR group (225 U/L vs. 198 U/L, *p* = 0.015) (**Table 1**). These results indicate that higher peripheral blood inflammatory ratios may be closely associated with aggressive disease features.

### Correlation with the peak levels of CAR transgene

**Figure 1:**
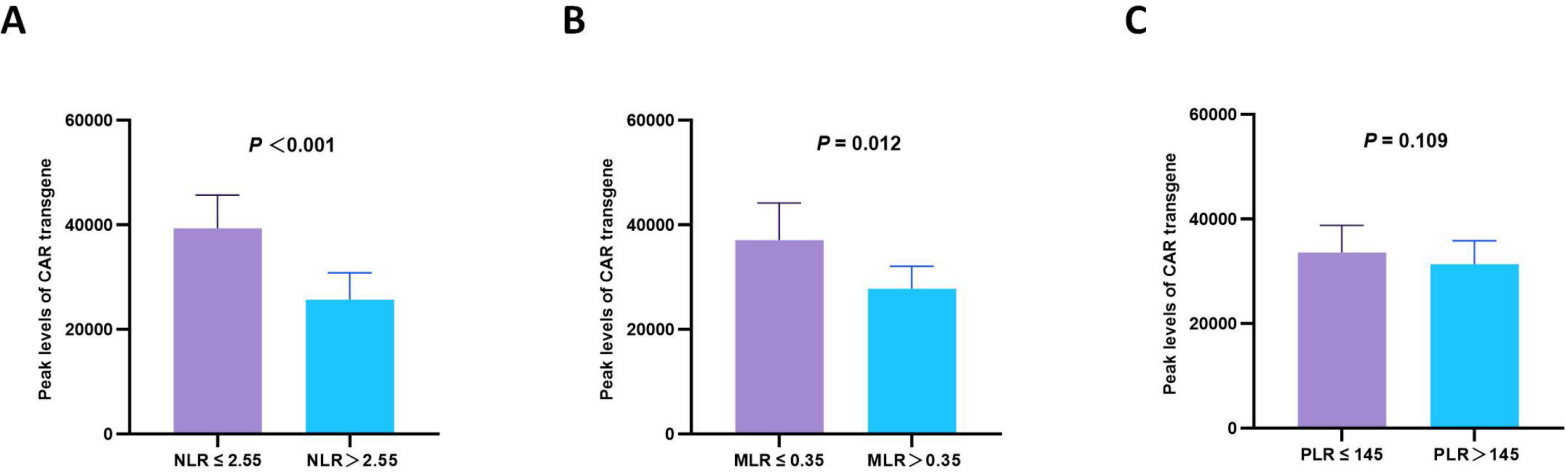
Subgroup analysis of the peak levels of CAR transgene. Box plots show the peak levels of CAR transgene (copies/μg DNA) in subgroups stratified by **(A)** NLR (*p* < 0.001), **(B)** MLR (*p* = 0.012), and **(C)** PLR (*p* = 0.109).

During dynamic monitoring post-T cell infusion, significant differences in the peak levels of CAR transgene were observed between different ratio groups. The peak levels of CAR transgene were significantly higher in the low NLR group compared to the high NLR group (*p* < 0.001). Similarly, the low MLR group exhibited a higher peak level of CAR transgene (*p* = 0.012), while no significant difference was observed between PLR groups (*p* = 0.109) (**Figures 1A–C**). This finding suggests that low peripheral blood inflammatory ratios may facilitate *in vivo* expansion of CAR-T cells.

### Correlation with objective response rate

**Figure 2:**
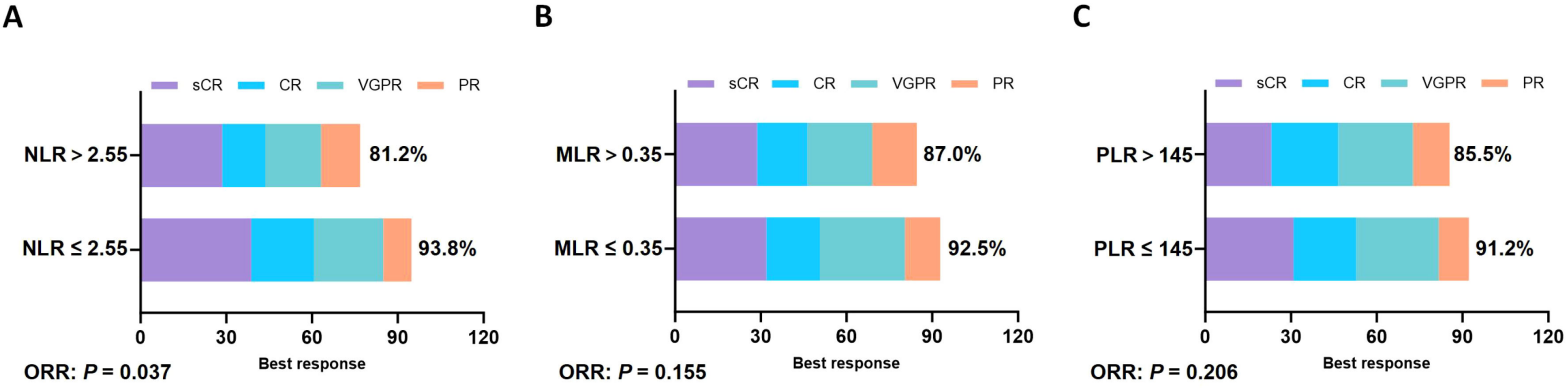
Subgroup analysis of ORR. Comparison of the better response and ORR between high and low subgroups of **(A)** NLR (≤ 2.55: 93.8% vs > 2.55: 81.2%; *p* = 0.037), **(B)** MLR (≤ 0.35: 92.5% vs > 0.35: 87.0%; *p* = 0.155), and **(C)** PLR (≤ 145: 91.2% vs > 145: 85.5%; *p* = 0.206).

The overall ORR for CAR-T therapy was 89.8%. Analysis by NLR groups indicated that in the low NLR group (≤ 2.55), 105 patients (93.8%) achieved a response, comprising 43 (38.4%) with stringent complete response (sCR), 21 (18.7%) with complete response (CR), 30 (26.8%) with very good partial response (VGPR), and 11 (9.8%) with partial response (PR). In contrast, the ORR in the high NLR group (> 2.55) was 81.2%, comprising 24 (27.8%) sCR, 14 (16.6%) CR, 19 (22.0%) VGPR, and 12 (14.1%) PR. The difference in ORR between these two groups was statistically significant (*p* = 0.037) (**Figure 2A**).

In the MLR groups, the ORR for the low MLR group (≤ 0.35) was 92.5%, comprising 38 (31.7%) sCR, 21 (17.5%) CR, 35 (29.2%) VGPR, and 17 (14.1%) PR. The ORR for the high MLR group (> 0.35) was 87.0%, comprising 22 (28.6%) sCR, 12 (15.6%) CR, 20 (25.9%) VGPR, and 13 (16.9%) PR. The difference in ORR between MLR groups had no statistical significance (*p* = 0.155) (**Figure 2B**).

In the PLR groups, the ORR for the low PLR group (≤ 145) was 91.2%, comprising 35 (30.7%) sCR, 20 (17.5%) CR, 33 (28.9%) VGPR, and 16 (14.1%) PR. The ORR for the high PLR group (> 145) was 85.5%, comprising 22 (26.5%) sCR, 16 (19.3%) CR, 20 (24.1%) VGPR, and 13 (15.7%) PR. The difference in ORR between PLR groups had no statistical significance (*p* = 0.206) (**Figure 2C**).

Overall, patients in the low NLR group not only had a higher ORR but also a significantly higher rate of deep responses (sCR + CR) compared with the high NLR group (57.1% vs. 44.4%, *p* = 0.019), suggesting that low peripheral blood inflammatory ratios may be associated with a strong CAR-T cell response and thorough tumor clearance.

### Correlation with CRS and ICANS

CRS and ICANS are unique inflammation-related complications of CAR-T cell therapy. In this study, the incidence of severe CRS (≥ grade 3) was significantly higher in the high NLR group compared with the low NLR group (20.0% vs. 7.1%, *p* = 0.009). No statistically significant differences were observed between MLR groups (*p* = 0.396) or PLR groups (*p* = 0.081) (**Table 2**). Analysis of inflammatory cytokines revealed that the peak levels of IL-6 and ferritin were significantly elevated in the high NLR, MLR, and PLR groups (all *p* < 0.05) (**Supplementary Table S2**). Furthermore, NLR displayed a significant positive correlation with both the peak levels of ferritin and CRP (r = 0.295, *p* = 0.015) (**Supplementary Table S3**). Although the peak levels of CRP tended to be higher in the high inflammatory ratio groups than that of the low inflammatory ratio groups, the inter-group difference was not statistically significant (*p* > 0.05). These results suggest that the high peripheral blood inflammatory ratios are closely relationship with the severity of CRS and the level of systemic inflammatory response.

**Table 2:** Association with CRS.

	Overall (n = 197)	NLR			MLR			PLR		
		≤2.55 (n = 112)	>2.55 (n = 85)	*P*	≤0.35 (n = 120)	>0.35 (n = 77)	*P*	≤145 (n = 114)	>145 (n = 83)	*P*
CRS grades				**0.009**			0.396			0.081
1-2	172	104 (92.9)	68 (80.0)		106 (88.3)	64 (83.1)		104 (91.2)	68 (81.9)	
≥3	25	8 (7.1)	17 (20.0)		14 (11.7)	13 (16.9)		10 (8.8)	15 (18.1)	

CRS, Cytokine release syndrome.

Bold type indicates statistical significance (p < 0.05).

A total of 8 patients (4.1%) experienced ICANS in the entire cohort, including 3 cases (1.5%) of ≥ grade 3 ICANS. No significant difference in ICANS incidence was observed across NLR (*p* = 0.539), MLR (*p* > 0.999), or PLR (*p* = 0.536) groups (**Supplementary Table S1**).

### Correlation with PFS

**Figure 3:**
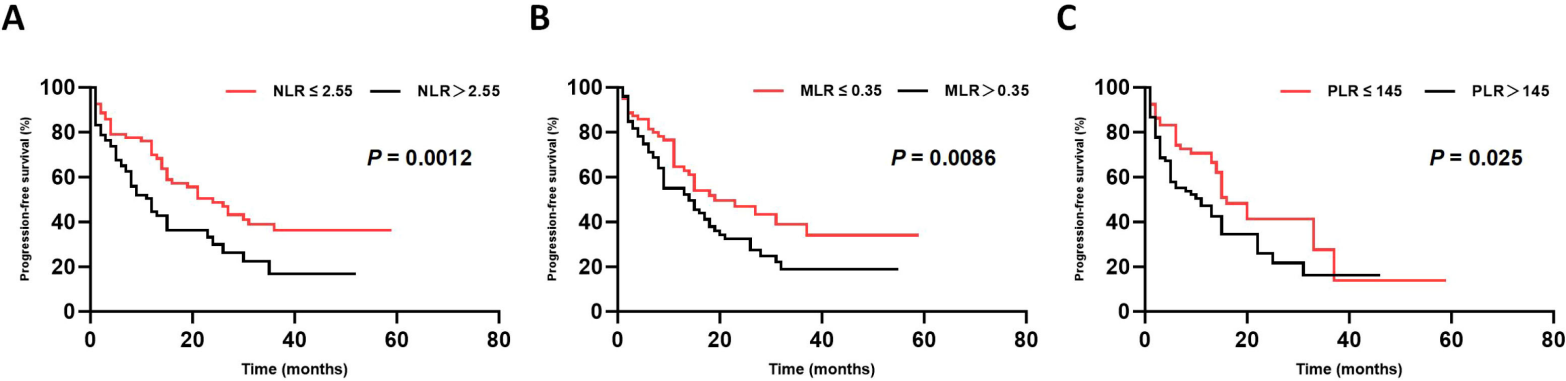
Subgroup analysis of PFS. Comparison of PFS between low and high subgroups of **(A)** NLR (*p* = 0.0012), **(B)** MLR (*p* = 0.0086), and **(C)** PLR (*p* = 0.025) using Kaplan-Meier analysis and log-rank tests. The low subgroup demonstrated superior PFS in all three comparisons.

Kaplan-Meier survival analysis showed that the PFS was significantly longer in the low NLR group compared to the high NLR group (median PFS: 18.6 months vs. 10.9 months, HR = 2.412, *p* = 0.0012) (**Figure 3A**). Similarly, the low MLR group exhibited longer PFS (median PFS: 16.3 months vs. 11.2 months, HR = 2.135, *p* = 0.0086) than that of the high MLR group (**Figure 3B**). Furthermore, PFS was also better in the low PLR group compared to the high PLR group (median PFS: 16.5 months vs. 13.4 months, HR = 1.825, *p* = 0.025) (**Figure 3C**).

**Figure 4:**
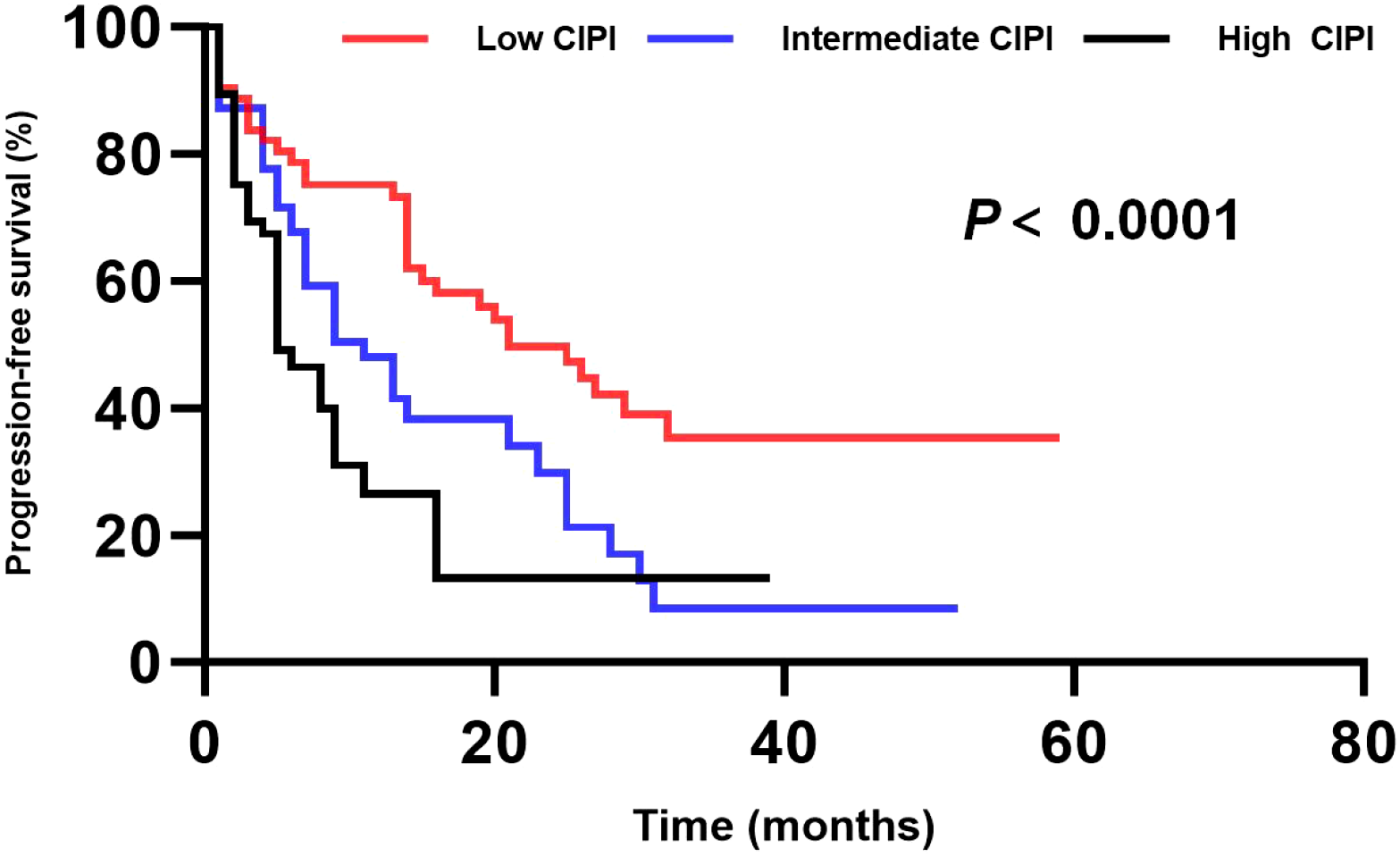
PFS stratified by the Cellular Inflammatory Prognostic Index (CIPI). Kaplan-Meier curves compare PFS among patients categorized into low-, intermediate-, and high-risk groups based on their CIPI score. A significant difference in PFS was observed across the three risk groups (log-rank test, *p* < 0.0001).

Based on the hazard ratios (HR) for NLR, MLR, and PLR groups, a Cellular Inflammatory Prognostic Index (CIPI) was constructed: both the high NLR and MLR were assigned 1 point, but the high PLR was assigned 0.5 point. Patients were categorized into three risk groups based on CIPI score: low-risk (0-0.5 points, 98 patients, 49.7%), intermediate-risk (1-1.5 points, 67 patients, 34.0%), and high-risk (2-2.5 points, 32 patients, 16.2%). PFS differed significantly among these three groups: median PFS was 18.9 months (95% CI: 13.8-25.1 months) for the low-risk group, 13.8 months (95% CI: 10.2-17.4 months) for the intermediate-risk group, and 5.1 months (95% CI: 2.7-7.5 months) for the high-risk group (*p* < 0.0001) (**Figure 4**).

To further assess whether CIPI is an independent prognostic factor for PFS, variables including age, sex, extramedullary disease, number of prior lines of therapy, MM subtype, high-risk cytogenetic status, R-ISS stage, tumor burden, previous infections, previous corticosteroid use, and CIPI score were incorporated into a Cox proportional hazards model. Univariate analysis identified R-ISS stage (p = 0.036), high tumor burden (p = 0.015), extramedullary lesions (p = 0.028), LDH level (p = 0.043), previous infections (p = 0.024), and CIPI score (p < 0.001) as the significant factors affecting PFS. These significant variables were subsequently included in the multivariate analysis, which revealed that the high tumor burden (p = 0.041) and CIPI score (p = 0.013) were independent risk factors for PFS in R/R MM patients received CAR-T cell therapy (**Table 3**).

**Table 3:** Univariate and multivariate analysis of PFS.

Variables	Progression-free survival (PFS)			
	Univariate analysis		Multivariate analysis	
	HR (95% CI)	*P*	HR (95% CI)	*P*
Age, >60 years	0.722 (0.544-1.341)	0.532		
Gender, Male	1.038 (0.713-2.13)	0.655		
R-ISS stage III	1.87 (1.301-2.386)	**0.036**	1.125 (0.566-1.89)	0.225
Type of myeloma, IgG	1.562 (0.417-1.891)	0.326		
Type of CAR-T cell therapy				
BCMA (reference)				
BCMA+CD19	1.266 (0.816-1.458)	0.745		
High tumor burden	2.583 (1.407-4.188)	**0.015**	1.355 (1.103-2.68)	**0.041**
High-risk cytogenetic features	1.824 (0.613-2.199)	0.39		
Extramedullary lesions	1.891 (1.162-3.092)	**0.028**	1.895 (0.827-2.233)	0.109
Previous therapy lines, ≥6	1.407 (0.655-1.983)	0.436		
Previous HCT	1.254 (0.717-1.784)	0.613		
LDH, >ULN	1.218 (1.083-3.188)	**0.043**	1.05 (0.652-1.855)	0.31
β2-MG, ≥5500ng/ml	1.388 (0.711-2.165)	0.314		
CIPI score		**<0.001**		**0.013**
Low (reference)				
Intermediate	2.365 (1.623-3.175)	**0.022**	2.02 (1.261-2.983)	**0.031**
High	4.17 (1.965-6.414)	**<0.001**	2.618 (1.307-4.205)	**0.02**
Previous infections	2.133 (1.816-3.103)	**0.024**	1.622 (0.811-2.115)	**0.075**
Previous corticosteroid use	1.439 (0.51-2.481)	0.103		

ULN, upper limit of normal; CIPI, Cellular Inflammatory Prognostic Index.

Bold type indicates statistical significance (p < 0.05).

## Discussion

With the growth of research on CAR-T cell therapy in R/R MM has substantiated its significant efficacy. However, many patients still experience disease progression or relapse soon after treatment, highlighting heterogeneity in immune response and prognosis among individuals. Therefore, identifying biomarkers capable of predicting CAR-T efficacy and related toxicities is crucial for optimizing patient selection and achieving personalized management. This study retrospectively analyzed data from 197 R/R MM patients who received CAR-T therapy. Using ROC curves, the optimal cut-off values for baseline NLR, MLR, and PLR and their relationships with clinical characteristics were determined. Our results suggested that low NLR, MLR, and PLR levels were significantly associated with high ORR and long PFS, with all three ratios being statistically significant in univariate analysis. The constructed CIPI score effectively stratified patients into distinct prognostic risk groups and was confirmed as an independent predictor of PFS in multivariate analysis.

Previous studies have indicated that NLR, MLR, and PLR served as indicators reflecting the host’s immune-inflammatory balance and were simple, reliable prognostic markers in various solid tumors and hematologic malignancies. An elevated NLR typically suggests enhanced neutrophil-mediated pro-inflammatory response coupled with impaired anti-tumor immune function reflected by lymphocytopenia (26). Increased MLR and PLR might be associated with monocyte- and platelet-mediated immune suppression, cytokine release, and tumor-promoting effects (27). Consequently, high peripheral blood inflammatory ratios often indicate a poor prognosis, consistent with the results of our study. Notably, in the baseline characteristic analysis, patients with high NLR and high MLR more frequently had higher tumor burden than that of patients with low NLR and MLR, while high PLR patients had a higher incidence of extramedullary lesions than that of patients with low PLR. Furthermore, LDH levels were higher in the high NLR group than that of patients with low NLR. The collinearity between these known adverse biological features and peripheral blood inflammatory ratios suggests that these inflammatory ratios may serve as surrogate markers for disease aggressiveness and potentially represent the host’s inflammatory-immune status independent of traditional risk factors, although the precise mechanisms require further investigation.

CRS is a common adverse reaction to CAR-T therapy. Our center previous studies implied that severe CRS was associated with poorer survival outcomes (28). Therefore, early identification of patients at risk for severe CRS is crucial. In this study, the incidence of ≥ grade 3 CRS was significantly higher in the high NLR and MLR groups than that of patients with low NLR and MLR. Elevated NLR and MLR reflect an increased proportion of neutrophils and monocytes related to lymphocytes, indicating a pro-inflammatory state and immune dysregulation (29). Activated neutrophils and monocytes can release large quantities of pro-inflammatory cytokines like IL-6, a key mediator in CRS pathogenesis. IL-6 induces hepatic synthesis of CRP and activates macrophages, thereby amplifying the inflammatory cascade (30, 31). Elevated ferritin levels imply intense activation of the monocyte-macrophage system and iron metabolism dysregulation, serving as sensitive indicators of high inflammatory burden and cytokine storm severity (32, 33). Previous studies reported that patients with high peak levels of ferritin, CRP, and IL-6 were much more prone to severe CRS than that of patient with low levels of ferritin, CRP, and IL-6 (15, 34, 35). Our further study suggested that the peak levels of ferritin and IL-6 were significantly elevated in the high NLR, MLR, and PLR groups, and correlation analysis displayed positive associations, providing biological plausibility for using peripheral blood inflammatory ratios to predict CRS.

Notably, Zhang et al. Implied that the low absolute neutrophil count in patients with B-cell malignancies was associated with better CAR-T cell expansion *in vivo* than that of patients with high absolute neutrophil count. The underlying mechanism might be involved in neutrophils suppressing T-cell function via the release of reactive oxygen species, proteases, or induction of myeloid-derived suppressor cells, thereby limiting CAR-T cell expansion and cytotoxicity (36–38). In our study, patients in low NLR and MLR groups had significantly high peak levels of CAR transgene, further supporting the notion that CAR-T cells achieve more effective expansion in a host environment with lower inflammatory burden and better-preserved lymphocyte counts than that of patients with high inflammatory burden and little preserved lymphocyte counts. The specific mechanisms warrant further investigation.

Furthermore, we constructed a comprehensive Cellular Inflammatory Prognostic Index (CIPI) based on these three inflammatory indicators. Our results demonstrated that patients in the high CIPI group had significantly shorter PFS than that of patients with low CIPI. Even after adjusting for traditional factors such as high tumor burden and extramedullary lesions, CIPI remained as an independent risk factor. Therefore, CIPI may serve as a simple, reproducible biomarker to help identify high-risk patients before treatment, informing personalized CAR-T regimen design and prophylactic interventions.

This study also has some limitations. First, as a single-center retrospective analysis, the sample size is limited, and in-depth subgroup analysis for different CAR-T products/targets was not performed. Second, while sensitivity analyses adjusted for common confounders (e.g., previous infection, corticosteroid use), the retrospective design precluded comprehensive data capture. Thus, unmeasured confounding remains possible, and the prognostic specificity of NLR, MLR, and PLR calls for validation in prospective cohorts with detailed baseline profiling. Additionally, although inflammatory indicators were significantly associated with survival outcomes, a causal relationship cannot be established. Future prospective studies and mechanistic experiments are needed to explore roles of these indicators in CAR-T cell expansion, exhaustion, and immune microenvironment remodeling.

## Conclusion

In summary, this study suggests that baseline peripheral blood inflammatory ratios are associated with the efficacy and toxicity of CAR-T therapy. The CIPI score, derived from these ratios, may serve as an independent prognostic factor of PFS in R/R MM patients. Incorporating these indicators into pre-treatment screening may provide a valuable basis for refining risk stratification and personalizing management strategies.

## Data Availability

The original contributions presented in the study are included in the article/supplementary material. Further inquiries can be directed to the corresponding authors.

## Supplementary material

Supplementary Table 1 Association with ICANS.

Supplementary Table 2 Association with inflammatory biomarkers.

Supplementary Table 3 Correlation analysis with inflammatory biomarkers.

Supplementary Figure 1 ROC analysis of NLR, MLR, and PLR for PFS prediction. The area under the curve (AUC) was 0.801 for NLR **(A)**, 0.763 for MLR **(B)**, and 0.755 for PLR **(C)** in 197 patients with R/R MM following CAR-T therapy (all *p* < 0.001).
