# Supplementary material for: Peripheral blood inflammatory ratios predict efficacy and toxicity of CAR-T cell immunotherapy in relapsed/refractory multiple myeloma
Source: Front Immunol. 2026 Feb 25;17:1752235. doi: 10.3389/fimmu.2026.1752235 (PMC12975872; doi:10.3389/fimmu.2026.1752235)
Supplement: Supplementary Table 1 — Association with ICANS. [file Table1.docx]

**Table S1.** Association with ICANS.

|  | **Overall**  **(n = 197)** | **NLR** | | | **MLR** | | | **PLR** | | |
| --- | --- | --- | --- | --- | --- | --- | --- | --- | --- | --- |
|  |  | **≤2.55**  **(n = 112)** | **＞2.55**  **(n = 85)** | ***P*** | **≤0.35**  **(n = 120)** | **＞0.35**  **(n = 77)** | ***P*** | **≤145**  **(n = 114)** | **＞145**  **(n = 83)** | ***P*** |
| **ICANS grades** |  |  |  | 0.539 |  |  | ＞0.999 |  |  | 0.536 |
| 0 | 189 (95.9) | 109 (97.3) | 80 (94.1) |  | 115 (95.8) | 74 (96.1) |  | 111 (97.4) | 78 (94.0) |  |
| 1-2 | 5 (2.5) | 2 (1.8) | 3 (3.5) |  | 3 (2.5) | 2 (2.6) |  | 2 (1.8) | 3 (3.6) |  |
| ≥3 | 3 (1.5) | 1 (0.9) | 2 (2.4) |  | 2 (1.7) | 1 (1.3) |  | 1 (0.8) | 2 (2.4) |  |

ICANS, immune effector cell-associated neurotoxicity syndrome.
