# Supplementary material for: Peripheral blood inflammatory ratios predict efficacy and toxicity of CAR-T cell immunotherapy in relapsed/refractory multiple myeloma
Source: Front Immunol. 2026 Feb 25;17:1752235. doi: 10.3389/fimmu.2026.1752235 (PMC12975872; doi:10.3389/fimmu.2026.1752235)
Supplement: Supplementary Table 2 — Association with inflammatory biomarkers. [file Table2.docx]

**Table S2.** Association with inflammatory biomarkers.

| **Variables** | **Overall**  **(n = 197)** | **NLR** | | | **MLR** | | | **PLR** | | |
| --- | --- | --- | --- | --- | --- | --- | --- | --- | --- | --- |
|  |  | **≤2.55**  **(n = 112)** | **＞2.55**  **(n = 85)** | ***P*** | **≤0.35**  **(n = 120)** | **＞0.35**  **(n = 77)** | ***P*** | **≤145**  **(n = 114)** | **＞145**  **(n = 83)** | ***P*** |
| **Peak ferritin levels, ng/mL, median (range)** | 458.4  (9.5-5000) | 436.0  (9.9-5000) | 472.5  (9.5-5000) | **0.018** | 449.2  (9.5-5000) | 469.5  (12.6-4900) | **0.042** | 438.4  (9.5-5000) | 466.7  (11.2-5000) | **0.021** |
| **Peak CRP levels, mg/L, median (range)** | 5.2  (0.2-251.9) | 4.5  (0.2-240.5) | 6.2  (1.6-251.9) | 0.093 | 4.8  (0.2-229.2) | 5.6  (1.3-251.9) | 0.311 | 4.8  (0.2-218.2) | 6  (1.9-251.9) | 0.155 |
| **Peak IL-6 levels, pg/mL, median (range)** | 6.5 (1-60) | 5.8 (1.8-55) | 7.6 (1-60) | **0.011** | 6.1 (1.6-60) | 7.2 (1-58) | **0.03** | 5.9 (1.6-58) | 7.2 (1-60) | **0.028** |

CRP, C-reactive protein; IL-6, interleukin-6.
