# Supplementary material for: Peripheral blood inflammatory ratios predict efficacy and toxicity of CAR-T cell immunotherapy in relapsed/refractory multiple myeloma
Source: Front Immunol. 2026 Feb 25;17:1752235. doi: 10.3389/fimmu.2026.1752235 (PMC12975872; doi:10.3389/fimmu.2026.1752235)
Supplement: Supplementary Table 3 — Correlation analysis with inflammatory biomarkers. [file Table3.docx]

**Table S3.** Correlation analysis with inflammatory biomarkers.

| **Variables** | **NLR** | | **MLR** | | **PLR** | |
| --- | --- | --- | --- | --- | --- | --- |
|  | ***Spearman r*** | ***P*** | ***Spearman r*** | ***P*** | ***Spearman r*** | ***P*** |
| **Peak ferritin levels** | 0.295 | **0.015** | 0.036 | 0.645 | 0.112 | 0.133 |
| **Peak CRP levels** | 0.219 | **0.023** | 0.093 | 0.517 | 0.076 | 0.34 |
| **Peak IL-6 levels** | 0.137 | 0.22 | 0.134 | 0.352 | 0.118 | 0.096 |
