# Supplementary figures and images for: Peripheral blood inflammatory ratios predict efficacy and toxicity of CAR-T cell immunotherapy in relapsed/refractory multiple myeloma
Source: Front Immunol. 2026 Feb 25;17:1752235. doi: 10.3389/fimmu.2026.1752235 (PMC12975872; doi:10.3389/fimmu.2026.1752235)

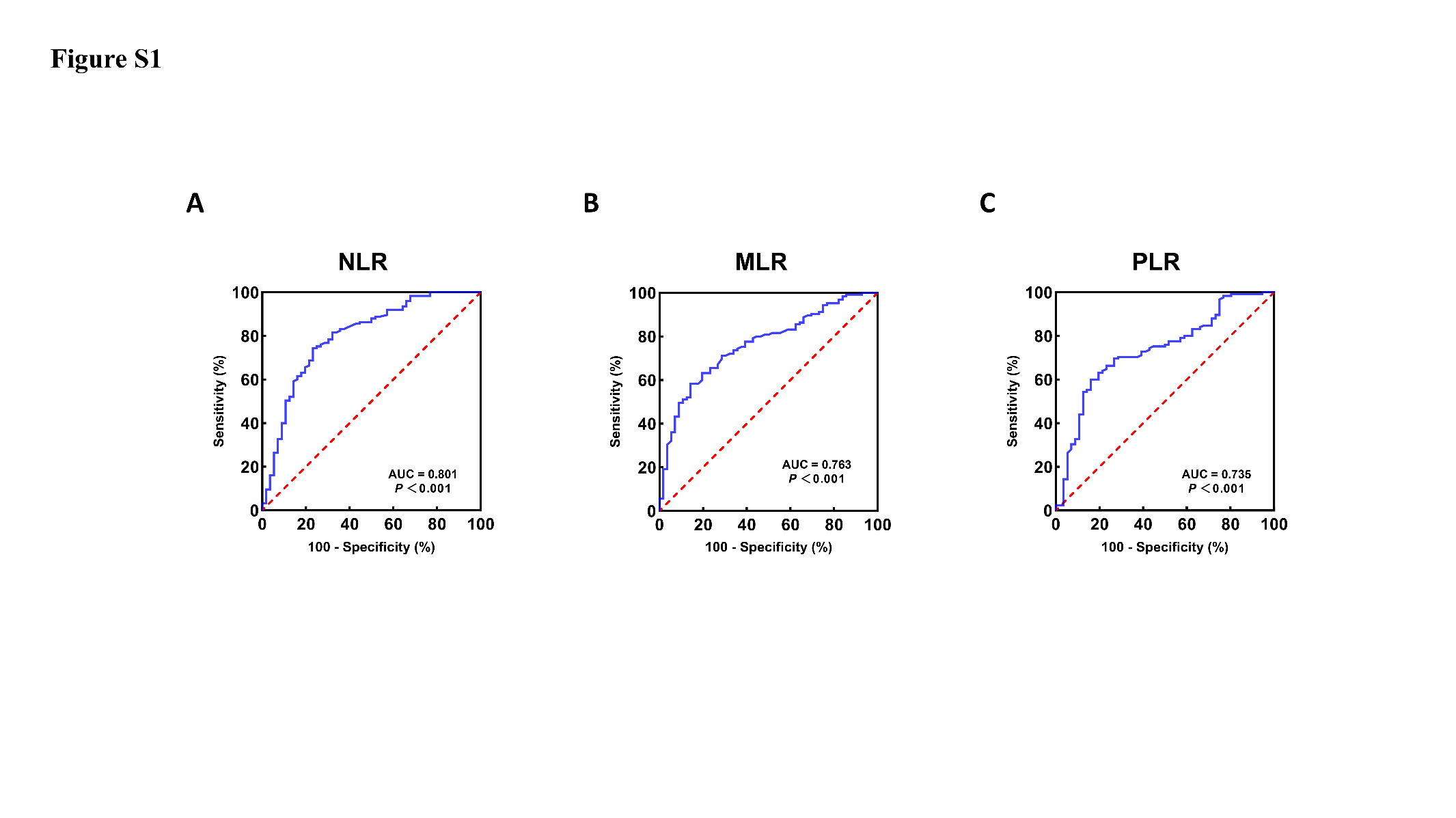

Supplement: Supplementary Figure 1 — ROC analysis of NLR, MLR, and PLR for PFS prediction. The area under the curve (AUC) was 0.801 for NLR (A), 0.763 for MLR (B), and 0.755 for PLR (C) in 197 patients with R/R MM following CAR-T therapy (all p < 0.001). [file DataSheet1.docx]
